# The TRKB rs2289656 genetic polymorphism is associated with acute suicide attempts in depressed patients: A transversal case control study

**DOI:** 10.1371/journal.pone.0205648

**Published:** 2018-10-11

**Authors:** Eric Deflesselle, Romain Colle, Laurent Rigal, Denis J. David, Albane Vievard, Séverine Martin, Laurent Becquemont, Céline Verstuyft, Emmanuelle Corruble

**Affiliations:** 1 INSERM UMR_S1178, Equipe “Dépression et Antidépresseurs”, Faculté de Médecine, CESP, Université Paris-Sud, Le Kremlin Bicêtre, France; 2 Département de Médecine Générale, Université Paris-Sud, Faculté de Médecine, Le Kremlin Bicêtre, France; 3 Service Hospitalo-Universitaire de Psychiatrie, Assistance Publique-Hôpitaux de Paris, Hôpitaux Universitaires Paris-Sud, Hôpital de Bicêtre, Le Kremlin Bicêtre, France; 4 INSERM UMR-S1178, Université Paris-Sud, Faculté de Pharmacie, CESP, Université Paris-Saclay, Chatenay-Malabry, France; 5 Service de Génétique Moléculaire, Pharmacogénétique et Hormonologie, Assistance Publique-Hôpitaux de Paris, Hôpitaux Universitaires Paris-Sud, Hôpital de Bicêtre, Le Kremlin Bicêtre, France; 6 Centre de Ressources Biologiques Paris Sud, Hôpital Bicêtre, Assistance Publique-Hôpitaux de Paris, Le Kremlin Bicêtre, France; Chiba Daigaku, JAPAN

## Abstract

**Introduction:**

Suicide Attempts (SA) are the main complications of Major Depressive Episodes (MDE) and are difficult to predict. Suicide is associated with the expression of Receptor Tyrosin-Kinase B (TRKB), the receptor of the Brain Derived Neurotrophic Factor (BDNF) involved in MDE. However, the impact of its genetic polymorphisms as predictive factors of SA should be clarified. Our main aim is to assess the association of 8 TRKB genetic polymorphisms and SA in depressed patients.

**Material and methods:**

In 624 patients currently experiencing an MDE in the context of Major Depressive Disorder (MDD) (METADAP study), we assessed the association between 8 TRKB genetic polymorphisms (rs1778933, rs1187352, rs2289658, rs2289657, rs2289656, rs3824519, rs56142442 and rs1439050) and acute (previous month) or past (older than one month) SA. Bonferroni corrections and multivariate analysis adjusted for age, sex, level of education, marital status, Hamilton Depression Rating Scale score and previous MDE were used.

**Results:**

The rs2289656 was associated with acute SA (CC = 28.5%, CT = 15.0% and TT = 11.5%, p = 0.0008). However, the other SNPs were not. Patients with the CC genotype had a higher rate of acute SA (28.5%) as compared to T carriers (14.6%) (adjusted OR = 2.2, CI95% [1.4; 3.5], p<0.0001).

**Conclusion:**

The TRKB rs2289656 CC genotype is associated with a 2.2 fold higher risk of acute SA in depressed patients. If this result could be confirmed, this *TRKB* SNP may be assessed to contribute to the prediction of SA in depressed patients.

## Introduction

Suicide Attempts (SA) are the main complications of Major Depressive Episodes (MDE). 15% of patients with MDE attempt suicide during their life [1]. Their risk of SA and of suicide death is respectively about 20 fold (odds ratio [OR] = 7.8–29.9) [2] and 6 fold higher than the general population [3]. Major Depressive Disorder (MDD) is the leading cause of SA and suicide deaths (40% of SA and 60% of the suicide deaths) [4]. The prediction of SA during MDE is difficult for clinicians and is a major public health issue. Thus, it could be useful to identify biomarkers that could improve the prediction of SA. Genetic biomarkers are of interest since heritability of suicidal behavior is high [5]. So, the purpose of this study is to identify genetic biomarkers of SA in depressed patients that may contribute to prevent SA in depressed patients.

The Tyrosine Kinase Receptor B (TRKB), which is the main receptor of the Brain Derived Neurotrophic Factor (BDNF), activates neuronal survival, plasticity, neurogenesis and synaptic connectivity [6]. BDNF is associated with major depressive episodes [7].

Interestingly, the BDNF/TRKB pathway and suicide may be linked. Indeed, TRKB and BDNF protein expression are decreased in the prefrontal cortex and hippocampus of suicide completers as compared to non-psychiatric healthy controls [8]. And an association between the BDNF Val66Met genetic polymorphism and SA was identified in a sample of 170 Italian depressed patients [9]. There were a lot of data involving BDNF and its main receptor TRKB in the neurobiology of suicide [10]. In depressed patients, most of the genetic studies regarding suicide attempts dealt with BDNF, whereas TRKB has been poorly explored [11].

Two recent post-mortem studies have investigated *TRKB* genetic polymorphisms in suicidal completers. The study performed by Ropret and colleagues did not find any association between 5 TRKB SNPs and suicide [12]. However, another one reported that 2 out of 17 TRKB SNPs (rs10868235 and rs1867283) were associated with suicide [13].

To the best of our knowledge, out of 4 studies comparing TRKB genetic polymorphisms in MDE patients with or without lifetime SA [14–17], 3 were negative. In the Schosser et al. study [17], comparing depressed cases and controls in a European cohort of 2023 patients, there was no association between TRKB SNPs and suicidal behavior. Similarly, no association between TRKB SNPs and lifetime suicide attempts was shown in the Perlis’ study [14] in the STAR*D American cohort of 1273 MDD and 3117 bipolar patients, and in the Mullins’ study [15] in a European cohort of 3270 bipolar and MDD patients. Only one study identified five TRKB SNPs out of 69 (rs10868235, rs1147198, rs1867283, rs1187286, and rs11140800) in German depressed patients [16]. This study found that there was a significant association with lifetime SA for these SNPs in two independent samples (a primary sample (n = 394) and a replication sample (n = 744)). Another case-control study in 159 psychiatric patients (all diagnoses included) reported a significant association between the TRKB rs1659400 SNP and lifetime SA in 71 females but not in males [18]. Four genetic studies have focused on suicidal ideation under antidepressant drug treatment. Voegeli et al. (2016) [19], in a case-control cohort of 3771 French depressed patients found a significant association between antidepressant-associated worsening of suicidal ideation and the rs1439050 but not 12 other TRKB SNPs. Perroud et al., 2009 [20], in a cohort of 811 European antidepressant-treated depressed patients reported an association between 4 TRKB SNPs (rs1187352, rs1778933, rs3824519, rs1439050) and antidepressant- associated worsening of suicidal ideation, that do not stand after correction for multiple comparisons. In the two other genome-wide association studies (GWAS), in two cohorts of 1953 European depressed patients treated with selective serotonin reuptake inhibitors (SSRI) and 706 European antidepressant-treated depressed patients, there was no evidence for an association between TRKB SNPs and antidepressant-associated worsening of suicidal ideation [21,22]. However, there are some inconsistencies that may be due to limits in the methodology related to study designs. Some of them [14–17] studied lifetime SA, assessed retrospectively through patient interviews. This methodology has two limits, first the memory bias related to delay between SA and assessment, and second the declarative bias of SA which are under-declared by patients [23–26]. Indeed, more than one third of patients with major depression misreport their history of SA [25]. Other studies [19,20] assessed suicidality prospectively using specific items of depression scales. But, in these studies, SA were not assessed and there was a discrepancy between individuals with suicide ideation and suicide attempters [27]. To overcome these limits, we proposed to study SA and more specifically acute SA in MDE patients with both self-assessment and assessments by professionals of mental health.

Furthermore, 8 TRKB SNPs (rs1778933, rs1187352, rs2289658, rs2289657, rs2289656, rs3824519, rs56142442 and rs1439050) were not previously or poorly explored and were potentially relevant.

Our primary objective was to compare 8 TRKB SNPs genotypes (rs1778933, rs1187352, rs2289658, rs2289657, rs2289656, rs3824519, rs56142442 and rs1439050) in patients with and without acute SA. Then, our secondary objective was to compare TRKB SNPs genotypes in patients with and without past SA.

## Material and methods

### Designs

This was a transversal case-control study, using the METADAP study [28], registered by the French National Agency for Medicine and Health Products Safety (ANSM) and the Commission Nationale de l'Informatique et des Libertés (CNIL), approved by the Ethics Committee of Paris-Boulogne, France, and conforming to international ethical standards. The association between 8 TRKB SNPs and suicide attempts were assessed in patients with a current MDE.

### Patients

Since suicidal behaviors may be multifactorial, we focused on a homogenous sample of patients with a current major depressive episode in a context of MDD. Patients were aged 18–65 years, with a current MDE diagnosis in a context of Major Depressive Disorder (MDD) (DSM-IVTR) based on the Mini International Neuropsychiatric Interview (MINI), with a minimum depression score of 18 on the17-item Hamilton Depression Rating Scale (HAMD) [29]. Patients with DSM-IVTR bipolar disorders, psychotic disorders, current substance abuse or dependence, pregnancy, breast feeding, organic brain syndromes or unstable medical conditions were excluded. Patients were provided written informed consent for study participation and for genetic analyses. Clinical assessments were performed blind to genotyping results.

### Suicide attempts

The main criterion was acute SA, defined by a self-destructive act with some intent to end one’s life which occurred in the month before the assessment [30]. Acute SA were assessed firstly through patient interviews with both a psychiatrist and a psychologist, and secondly were screened in the medical records. This strategy using both self-assessment and assessment by others was chosen to overcome memory bias and declarative bias. The secondary criterion was past SA, defined as those occurring more than one month before assessment. Past SA were assessed by patient interviews with both a psychiatrist and a psychologist. Past SA were chosen as secondary criteria because they suffer particularly from memory and declarative biases.

### Genotype

We focused on SNPs which were not previously or poorly explored and that were potentially relevant. 5 SNPs (rs1778933, rs1187352, rs3824519 rs1439050 and, rs2289656) were identified for their potential association with suicidality in previous studies [16,19,20]. One of those SNP (rs2289656) was associated with Alzheimer disease [31]. We also assessed three SNPs never assessed with SA (rs2289658, rs2289657, rs56142442), because the Dong’s study showed that they have a possible impact on the efficacy of antidepressant in patients with MDD [32]. Six of them were introns and the two others were synonymous exons (S1 Table).

Genomic DNA was extracted from circulating blood leucocytes by using Gentra Puregene Blood Kits according to the manufacturer’s protocol (Qiagen) and was stored at -20°C.

The TRKB polymorphisms were genotyped by the IntegraGen company (Evry, France) using TaqMan allelic discrimination [33](dx.doi.org/10.17504/protocols.io.ti2ekge).

### Statistical analysis

The statistical analysis was performed with the R 3.2.2 software. Linkage disequilibrium (LD) between the 8 SNPs was assessed using THESIAS 3.1 software [34]. After testing the Hardy-Weinberg equilibrium using Chi 2 tests, each SNP was studied in 3 groups (two homozygote and one heterozygote groups). Regarding SNP with a significant association in 3 groups, we performed a complementary analysis in two groups (the heterozygote group being associated with one of the homozygote groups to test dominant and recessive models). We performed a complementary allelic analysis.

Due to the number of SNPs studied and to take into account the increased risk of false positive results due to the high number of comparisons, Bonferroni’s corrections were applied, the threshold for significance being 0.05/8 = 0.00625.

We performed bivariate analyses to assess associations between TRKB SNPs genotypes (and alleles) and socio-demographic clinical variables. Then, for the primary and secondary objectives, inter-genotypic comparisons were computed using Chi2 for acute SA and past SA. Logistic regressions were performed to control for potential confounders among demographic and clinical variables. Indeed, covariables of the multivariate models were selected on the basis of a significant association (p<0.05) in bivariate analyses with acute SA, past SA or SNPs. Adjustments were performed on age, sex, level of education, marital status, HAMD score, previous antidepressant treatment and previous MDE.

Due to the heterogeneity of the sample, the previous analysis was performed first in the whole sample and second in the Caucasian (patient with 2 Caucasian parents) subgroup of patients.

## Results

### Sample characteristics

Among the 624 depressed patients of the METADAP study, only 569 patients had a DNA sample. They were mainly women (69.6%). Their mean age was 46.2 years (sd = 13.2). Their mean HAMD score at baseline was 24.6 (sd = 5.0). Five hundred and twenty-one (92%) patients were Caucasian.

### Suicide attempts

22.8% of patients had an acute SA. 50.8% of patients with acute SA had a past SA. Socio-demographical and clinical characteristics of these patients are shown in S2 Table. Patients with acute SA were younger, had higher HAMD scores as compared to patients without acute SA. There was no significant difference in the other demographic and clinical features. Patients with past SA had a higher frequency of recurrent MDE and previous antidepressant drug treatment. There was no significant difference in the other demographic and clinical features.

### TRKB genetic polymorphisms

S3 Table showed the allelic repartition of the eight *TRKB* SNPs. Since there were no homozygous patients for the minor allele and only three heterozygous patients for the rs56142442, this SNP was not analyzed. There was a deviation from the Hardy–Weinberg equilibrium for two other SNPs (rs2289658 and rs3824519). The haplotype analysis (S4 Table) showed that rs1439050, rs1187352 and rs1778933 were in moderate linkage disequilibrium and that rs2289658, rs2289657, rs2289656 and rs3824519 were in high linkage disequilibrium.

There was no significant difference between the 8 TRKB genetic polymorphisms in the demographic and clinical features after Bonferroni correction (in the 3 group analysis and in the complementary 2 group analysis) except for educational level (p = 0.001 with rs1439050, p = 0.003 with rs1187352). The demographic and clinical features of the cohort according to each SNP are described in Table 1. The genotype groups did not significantly differ for these demographic and clinical features after Bonferroni corrections except educational level for rs1439050 and rs1187352.

**Table 1:** Demographic and clinical features according to the TRKB genetic polymorphisms studied.

	rs1439050			rs1187352			rs1778933			rs2289658			rs2289657			rs2289656			rs3824519		
Genotype	GG	GT	TT	GG	GA	AA	TT	TC	CC	AA	AG	GG	GG	GT	TT	CC	CT	TT	CC	CT	TT
Patients (n)	194	265	73	266	223	59	280	215	56	504	39	4	514	37	2	340	181	26	468	75	9
Caucasian patients (n)	190	236	60	243	204	56	252	196	55	467	31	2	475	29	2	307	169	25	432	65	8
Age (m(sd))	45 (13.3)	46.5 (13.1)	49 (13.0)	45.7 (12.8)	46.4 (13.6)	47.9 (12.6)	45.7 (12.7)	47.4 (13.5)	44.7 (13)	46.2 (13.3)	45.6 (11.3)	58.1 (1.7)	46.2 (13.2)	47.3 (11.2)	59.5 (0.3)	45.1^e^ (13.6)	48.3^e^ (12.0)	47.9^e^ (12.2)	46.4 (13.3)	45.3 (11.5)	47.2 (14.2)
Women (%(n))	71.6 (139)	69.4 (184)	68.5 (50)	69.9 (186)	71.7 (160)	64.4 (38)	68.6 (192)	72.6 (156)	69.6 (39)	71.4 (360)	59 (23)	50 (2)	70.6 (363)	62.2 (23)	0 (0)	69.7 (237)	69.1 (125)	84.6 (22)	70.3 (329)	68 (51)	55.6 (5)
Single (%(n))	51.5 (100)	51.3 (136)	60.3 (44)	56.4 (150)	48.4 (108)	57.6 (34)	51.1 (143)	51.6 (111)	66.1 (37)	52.2^d^ (263)	64.1^d^ (25)	0^d^ (0)	52.7 (271)	56.8 (21)	0 (0)	57.1 (194)	44.8 (81)	53.8 (14)	52.4 (245)	57.3 (43)	22.2 (2)
High educational level (%(n))	53.1^a^ (103)	36.2^a^ (96)	47.9^a^ (35)	61^b^ (125)	37.7^b^ (84)	47^b^ (36)	57.1^c^ (129)	39.1^c^ (84)	46.1^c^ (32)	44.2 (223)	53.8 (21)	0 (0)	44.4 (228)	51.4 (19)	0 (0)	47.9 (163)	39.8 (72)	30.8 (8)	43.8 (205)	49.3 (37)	11.1 (1)
Smoking (%(n))	41.8 (81)	34.7 (92)	32.9 (24)	40.7 (111)	30 (67)	41.7 (24)	39.3 (108)	33 (71)	38.6 (22)	36.1 (182)	43.6 (17)	0 (0)	36.2 (186)	45.9 (17)	0 (0)	35 (119)	35.9 (65)	61.5 (16)	35.7 (167)	45.3 (34)	33.3 (3)
Recurrent MDD (%(n))	76.2 (147)	74 (196)	75.3 (55)	73.6 (195)	75.3 (168)	81.4 (48)	71.7 (200)	78.1 (168)	78.6 (44)	75.5 (380)	74.4 (29)	50 (2)	75 (385)	73 (27)	100 (2)	77.1 (262)	70 (126)	84.6 (22)	74.7 (349)	76 (57)	77.8 (7)
HAMD-17 (m(sd))	24.2 (5.0)	24.6 (4.7)	24.9 (5.3)	24.3 (4.8)	24.7 (5.0)	25 (4.8)	24.6 (5.0)	24.3 (4.8)	25 (4.9)	24.5 (4.9)	25.1 (5.5)	23 (3.6)	24.5 (4.9)	24.7 (5.7)	26 (1.4)	24.5 (5.1)	24.4 (4.8)	24.8 (3.3)	24.5 (4.8)	24.2 (5.7)	25.8 (3.6)
antidepressant drug free (%(n))	41,2 (80)	34,3 (91)	38,4 (28)	39,5 (105)	32,7 (73)	42,4 (25)	40 (112)	31,6 (68)	44,6 (25)	36,9 (186)	41 (16)	25 (1)	37,4 (192)	32,4 (12)	50 (1)	39,1 (133)	34,3 (62)	30,8 (8)	37,2 (174)	34,7 (26)	44,4 (4)
Previous antidepressant treatment (%(n))	77.7 (150)	79.6 (211)	76.7 (56)	74.7 (198)	83.4 (186)	78 (46)	74.6 (208)	84.2 (181)	76.8 (43)	79.1 (398)	71.8 (28)	100 (4)	78.6 (403)	78.4 (29)	100 (2)	77.1 (262)	80 (144)	88.5 (23)	78.6 (367)	80 (60)	77.8 (7)
Suicide Attempt																					
Past SA (%(n))	30.4 (59)	32.5 (86)	30.1 (22)	32 (85)	28.7 (64)	40.7 (24)	29.3 (82)	32.6 (70)	41.1 (23)	31.9 (161)	30.8 (12)	50 (2)	31.9 (164)	29.7 (11)	100 (2)	33.2 (113)	29.8 (54)	26.9 (7)	30.8 (144)	36 (27)	44.4 (4)
Acute SA (%(n))	23.3 (45)	20.4 (54)	28.8 (21)	27.1 (59)	23.3 (52)	22.3 (16)	26.8 (66)	21.9 (47)	23.7 (15)	23.1 (116)	25.6 (10)	0 (0)	23.4 (120)	21.6 (8)	0 (0)	28.5 (97)	15 (27)	11.5 (3)	22.7 (106)	24 (18)	22.2 (2)

*n*: *number of patient; m*: *mean; sd*: *standard deviation; HAMD-17*: *Hamilton Depression Rating Scale 17 items; antidepressant drug free*: *absence of antidepressant* during 3 years before the inclusion; *previous antidepressant treatment*: *past history of antidepressant treatment; ant drug free in the 3 year before inclusion; MDD*: *Major Depressive Disorder; SA*: *Suicide Attempt; Past SA*: *Suicide attempts were defined as those which occurred more than one month before assessment*.

^a^: p = 0.001 (bivariate analysis) for inter-genotypic comparisons (3 groups)

^b^: p = 0.003 (bivariate analysis) for inter-genotypic comparisons (3 groups)

^c^: p = 0.039 (bivariate analysis) for inter-genotypic comparisons (3 groups)

^d^: p = 0.027 (bivariate analysis) for inter-genotypic comparisons (3 groups)

^e^: age (p = 0.025 (bivariate analysis) for inter-genotypic comparisons (3 groups)

### TRKB genetic polymorphisms and suicide attempts

There was an association between the rs2289656 and acute SA (bivariate: p = 0.0008 and adjusted: p = 0.003) (Table 1). The rs2289656 CC genotype, the major homozygote allele, was associated with higher rates of acute SA, as compared to CT (OR = 2.3, CI95% [1.4–3.7], adjusted OR = 2.1, CI95% [1.3–3.5]) and TT genotypes (OR = 3.1, CI95% [1.03 13.1], adjusted OR = 3, CI95% [1–12.9]). There was no association between rs2289656 and past SA. No significant association was observed with the 7 other SNPs.

**Fig 1:**
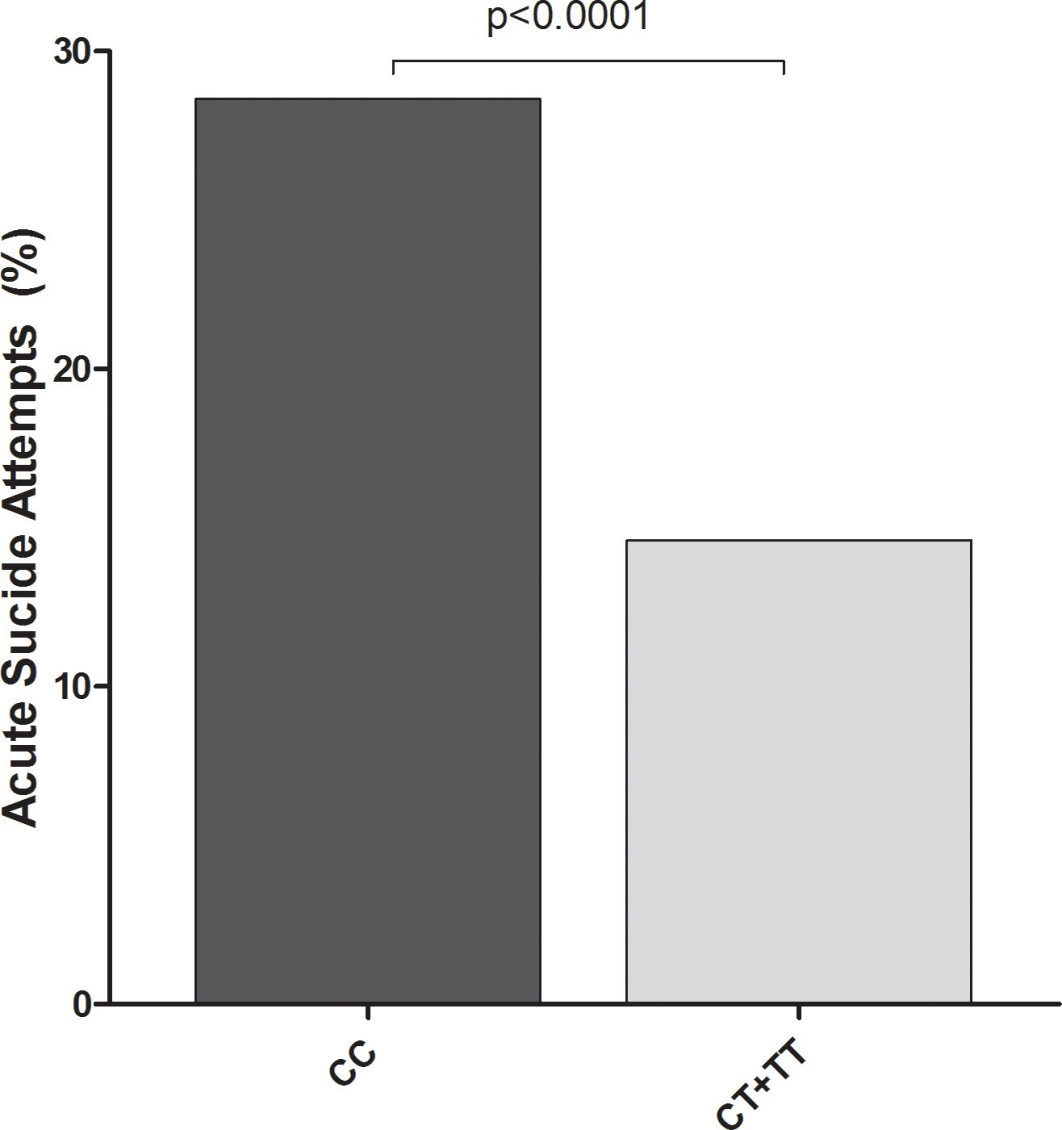
Association between the TRKB rs2289656 and acute suicide attempts. CC homozygotes versus (CT heterozygotes and TT homozygotes), p: bivariate p values.

In the 2-group analysis, a stronger association between rs2289656 and acute SA was observed (adjusted p = 0.001). Indeed, patients with the CC genotype had a higher rate of acute SA (28.5%) as compared to T carriers (14.6%) (OR = 2.3, CI95% [1.5; 3.7], adjusted OR = 2.2, CI95% [1.4–3.5]) (Fig 1). There was no association between rs2289656 and past SA. There was no significant association for the other SNPs.

The allelic analysis confirmed the association between rs2289656 and acute SA (bivariate: p = 0.0002; adjusted: p = 0.001). Indeed, patients with the C allele had a higher rate of acute SA (25.7%) than T carriers (14.2%) (OR = 2.1, CI95% [1.4–3.2], adjusted OR = 2.0, CI95% [1.3–3.0]) (S5 Table). There was no association between SNPs and past SA. There was no significant association for the other SNPs.

### TRKB genotype and suicide attempts in Caucasian patients

Similar results were found in the Caucasian subgroup (n = 521) with an association between the rs2289656 and acute SA (bivariate: p = 0.002 and adjusted p = 0.005). The CC genotype of the rs2289656 was associated with higher acute SA rates (CC: 29%, CT: 16.1%, TT: 12%) as compared respectively to CT (OR = 2.1, CI95% [1.3–3.5], adjusted OR = 2.1, CI95% [1.3–3.5]) and TT (OR = 3.0, CI95% [1.0–12.9], adjusted OR = 3, CI95% [1–13]) genotypes. There was no association between rs2289656 and past SA. No significant association was observed for the other 7 SNPs.

In the 2-group analysis, there was a stronger association between the rs2289656 and acute SA (bivariate: p = 0.00059, adjusted: p = 0.002). Patients with the CC genotype had a higher risk of acute SA as compared to T carriers (29% vs 14,6%, OR = 2.2 (CI95% [1.3; 3.6], adjusted OR = 2.1, CI95% [1.3–3.4]). There was no association between rs2289656 and past SA. There was no association for the other SNPs.

The allelic analysis confirmed the association between rs2289656 and acute SA (bivariate: p = 0.0007; adjusted: p = 0.002). Indeed, the C allele carriers had a higher rate of acute SA (26.2%) than the T allele carriers (15.1%) (OR = 2.0, CI95% [1.3–3.0], adjusted OR = 1.9, CI95% [1.3–2.9]). There was no association between rs2289656 and past SA and there was no significant association for the other SNPs.

### Haplotype

The haplotype rs2289658, rs2289657, rs2289656 and rs3824519 with high linkage disequilibrium, were associated with acute SA. Patients with the AGCC genotype had a higher rate of acute SA than AGTC genotype carriers (OR = 2.2 (CI95% [1.4; 3.2]), p = 0.00018).

## Discussion

The present study showed an association between the rs2289656 *TRKB* genetic polymorphism and acute SA in depressed patients. Indeed, patients with the CC genotype had 2.3 fold higher risk of acute SA as compared to T allele carriers. This SNP was not associated with MDD clinical features and remained significantly associated with acute SA after adjustment on MDD clinical and socio-demographical characteristics. Interestingly, in Kohli’s study [16], rs2289656 was not in linkage disequilibrium with the five TRKB SNPs significantly associated with lifetime SA. The rs2289656 was associated with Alzheimer disease (CC genotype is 3-fold more frequent in sporadic Alzheimer’s disease than the CT genotype) [31]. Some studies show an association between suicide attempts and the neurogenesis pathway genes such as BDNF, of which TRKB is the main receptor [11]. The neurotrophin genes (BDNF, NGF and TRKB) have the strongest evidence for a role in SA [35]. As depression is associated with neurotrophins [36], it could potentiate the effect of genetics on suicide attempts. A previous study [16] found a trend (p = 0.11) between this rs2289656 and lifetime SA in a German MDD sample (n = 394). Here, we show a significant association between this SNP and acute SA in a larger French MDD-MDE cohort. There were previous published GWAS of suicide attempts that did not catch TRKB SNPs [11,14,15,17,35,37,38]. Considering the large number of SNPs studied, in order to have sufficient statistical power, at least ten thousands patients should have been included [39]. Actually, these previous GWAS on SA did not have this sample size.

There were two published GWAS of lifetime SA in non MDD patients. They included bipolar patients (n = 5815 and n = 2698) [14,40] and they did not catch any associations between TRKB SNPs and lifetime SA. Thus, it could be suggested that the association between TRKB rs2289656 and acute SA observed in our study could be specific of MDE patients. However, regarding the very high number of tested SNPs (2 millions in the first one and 724 0000 in the second one), these two GWAS may have lacked power to conclude to the absence of association between rs2289656 and lifetime SA in bipolar disorder.

The rs1439050 was associated with antidepressant-worsening suicidal ideation in two studies [19,20]. In our study, in line with the replication cohort of Kholi [16], we failed to provide evidence for an association with acute SA. The discrepancy between Perroud or Voegeli and our results could be explained by the discrepancy between the main variable studied: suicidal ideation [19,20] and suicide attempt in our study. Indeed, most patients with suicide ideation do not make suicide attempts [27]. The rs1778933, rs1187352, rs3824519 were associated with antidepressant-associated worsening of suicidal ideation but not after Bonferroni correction in the study of Perroud et al., [20]. Here, no association between these SNPs and acute SA was observed.

This study had some limits. Firstly, the rs2289656 was an intron and its biological effect remains unknown. Some introns have a role in the mechanism of splicing and can change the mRNA transcript or its amount [41] and are involved in several human diseases [42]. Interestingly, a study reported that introns had an impact on TRKB splicing [43].

Because no control group without MDE was available, we were not able to assess the specific effect of MDE on the association between TRKB rs2289656 and acute SA. We cannot exclude masking effects of MDE. And the generalizability of the association between TRKB rs2289656 and acute SA cannot be proposed for other mental disorders or for the general population. However, as MDE is the leading cause of SA [4], our results are particularly relevant in this subpopulation. No association between the rs2289656 and past SA was observed. This suggests that past SA assessment may suffer from biases related to the delay between past SA and assessment, and the under-declared past SA by patients [23–26]. These biases are inherent to retrospective assessments of past events. We decided to limit the number of SNPs to avoid false positives and focus on potentially relevant and poorly explored SNPs. So, in this study, we did not include the five Kohli’s SNPs [16]. Finally, the effect size of rs2289656 impact is moderate (relative change 48.8%) and could partially predict acute SA in depressed patients. So, considered individually, the rs2289656 alone is not sufficient to predict acute SA but may contribute to this prediction. The total number of SA was not available in this study, because of the poor quality of such retrospective data collection in depressed patients because of cognitive symptoms. So, the associations between TRKB SNPs and total number of SA were not assessed. This study needs to be replicated in an independent cohort of MDE patients before introducing this biomarker in clinical assessments that could improve the accuracy of acute SA prediction.

This study has strengths. The sample size was large. The sample was well characterized and homogenous in terms of diagnosis (MDD patients with a current MDE only), with a majority of Caucasian patients. Furthermore, acute SA is a strong criterion: it is assessed by auto-evaluation, and evaluation by others, and medical records.

The BDNF/TRKB pathway has an influence on suicidality. There are epigenetic changes of BDNF and TRKB receptors in the brain of suicide patients [44] and Sarchiapone et al found an association between BDNF genetic polymorphisms and SA [9]. Although the functional and neurobiological significance of TRKB rs2289656 is poorly known, it has been shown to be associated with Alzheimer disease progression [31] suggesting that this SNP could be relevant. Since it is an intronic genetic polymorphism, it could play a role on alternative splicing. But further research is needed to understand the neurobiological significance of TRKB rs2289656 genetic polymorphism. To explore the generalizability of the association between TRKB rs2289656 and acute SA, future studies are needed in samples without MDE. These results need to be confirmed in future studies.

## Conclusion

This study in depressed patients showed an association between the rs2289656 *TRKB* SNP and acute SA. If it could be confirmed, there could be a benefit to genotype this *TRKB* SNP in daily practice to predict SA in depressed patients.

## Supporting information

S1 Table Studied SNPs description. (DOCX)

S2 Table Demographic and clinical features according to acute and past suicide attempts. (DOCX)

S3 Table Hardy Weinberg and allelic repartition. (DOCX)

S4 Table Linkage disequilibrium for the TRKB genetic polymorphisms studied. (DOCX)

S5 Table Demographic and clinical features according to the TRKB alleles. (DOCX)
