# Supplementary material for: The TRKB rs2289656 genetic polymorphism is associated with acute suicide attempts in depressed patients: A transversal case control study
Source: PLoS One. 2018 Oct 11;13(10):e0205648. doi: 10.1371/journal.pone.0205648 (PMC6181406; doi:10.1371/journal.pone.0205648)
Supplement: S1 Table — (DOCX) [file pone.0205648.s001.docx]

**S1 Table: Studied SNP description**

| SNP | Position | fonction |  | A1 | A2 | Gene | Freq A1 | Freq A2 |
| --- | --- | --- | --- | --- | --- | --- | --- | --- |
| rs1439050 | 87288192 | intron |  | G | T | NTRK2 | 0,673 | 0,327 |
| rs1187352 | 87293456 | intron |  | A | G | NTRK2 | 0,358 | 0,642 |
| rs1778933 | 87324410 | intron |  | C | T | NTRK2 | 0,35 | 0,65 |
| rs2289658 | 87563369 | exon syn |  | A | G | NTRK2 | 0,96 | 0,04 |
| rs2289657 | 87563459 | intron |  | G | T | NTRK2 | 0,942 | 0,058 |
| rs2289656 | 87563561 | intron |  | C | T | NTRK2 | 0,832 | 0,168 |
| rs3824519 | 87570003 | intron |  | C | T | NTRK2 | 0,916 | 0,084 |
| rs56142442 | 87636264 | exon syn |  | C | T | NTRK2 | 0,949 | 0,051 |

Position: on the chromosome 9 ; A1/2 : Allele 1/2; syn : synonym ; Feq : allelic frequency according to the SNPdb.
