# Supplementary material for: The TRKB rs2289656 genetic polymorphism is associated with acute suicide attempts in depressed patients: A transversal case control study
Source: PLoS One. 2018 Oct 11;13(10):e0205648. doi: 10.1371/journal.pone.0205648 (PMC6181406; doi:10.1371/journal.pone.0205648)
Supplement: S2 Table — (DOCX) [file pone.0205648.s002.docx]

**S2 Table: Demographic and clinical features according to acute and past suicide attempts.**

|  | Acute SA | | | Past SA | | |
| --- | --- | --- | --- | --- | --- | --- |
|  | no | yes | p | no | yes | p |
| Patients (%(n)) | 77  (438) | 22.8  (130) |  | 65.5  (372) | 34.5  (196) |  |
| Age (m(sd)) | 47.1  (12.7) | 43  (13.8) | 0.002 | 45.5  (13.5) | 46.2  (12.4) | 0.55 |
| Women (%(n)) | 68.3  (299) | 73.8  (96) | 0.22 | 66.9  (249) | 74.5  (146) | 0.06 |
| Single (%(n)) | 50.9  (223) | 56.9  (74) | 0.23 | 52.2  (194) | 52.6  (103) | 0.93 |
| High educational level (%(n)) | 43.8  (192) | 47.7  (62) | 0.44 | 45.0  (167) | 44.4  (87) | 0.67 |
| Smoking (%(n)) | 37  (162) | 35.4  (46) | 0.74 | 34.9  (130) | 39.8  (78) | 0.51 |
| Recurrent MDD (%(n)) | 74.9  (328) | 74.6  (97) | 0.95 | 65.3  (243) | 92.9  (182) | <0.0001 |
| Antidepressant drug free (%(n)) | 37.2  (158) | 40.6  (52) | 0.48 | 39.3  (143) | 35.4  (67) | 0.38 |
| Previous antidepressant treatment (%(n)) | 80.1  (351) | 73.1  (95) | 0.09 | 75.0  (279) | 85.2  (167) | 0.005 |
| HAMD (m(sd)) | 24.2  (4.9) | 25.9  (4.9) | 0.001 | 24.5  (5.0) | 24.8  (5.0) | 0.41 |

*n: number of patient; m: mean; sd: standard deviation; HAMD-17: Hamilton Depression Rating Scale 17 items; antidepressant drug free: no antidepressant since 3 years before assessment; previous antidepressant treatment: past history of antidepressant treatment; MDD: Major Depressive Disorder; SA: Suicide Attempt; Past SA: Suicide attempts were defined as those which occurred more than one month before assessment.*
