# Supplementary material for: The TRKB rs2289656 genetic polymorphism is associated with acute suicide attempts in depressed patients: A transversal case control study
Source: PLoS One. 2018 Oct 11;13(10):e0205648. doi: 10.1371/journal.pone.0205648 (PMC6181406; doi:10.1371/journal.pone.0205648)
Supplement: S3 Table — (DOCX) [file pone.0205648.s003.docx]

**S3 Table: Hardy Weinberg and allelic repartition**

| SNP | Ref | Alt | Ref H | Hz | Alt H | Chi2 | p |
| --- | --- | --- | --- | --- | --- | --- | --- |
| rs1778933 | T | C | 278 | 214 | 54 | 1.8 | 0.18 |
| rs1187352 | G | A | 266 | 221 | 59 | 1.6 | 0.20 |
| rs2289658 | A | G | 501 | 39 | 4 | 9.6 | 0.002 |
| rs2289657 | G | T | 512 | 37 | 2 | 2.2 | 0.14 |
| rs2289656 | C | T | 339 | 180 | 26 | 0.11 | 0.74 |
| rs3824519 | C | T | 467 | 74 | 9 | 6.48 | 0.01 |
| rs56142442 | C | T | 549 | 3 | 0 | 0.004 | 0.94 |
| rs1439050 | G | T | 194 | 262 | 73 | 1.1 | 0.3 |

Ref: Reference allele; Alt: Alternative Allele; H: Homozygote, Hz: Heterozygote.
