# Supplementary material for: The TRKB rs2289656 genetic polymorphism is associated with acute suicide attempts in depressed patients: A transversal case control study
Source: PLoS One. 2018 Oct 11;13(10):e0205648. doi: 10.1371/journal.pone.0205648 (PMC6181406; doi:10.1371/journal.pone.0205648)
Supplement: S4 Table — (DOCX) [file pone.0205648.s004.docx]

**Table S4: Linkage disequilibrium for the NTRK2 genetic polymorphisms studied.**

|  | rs1439050 | rs1187352 | rs1778933 | rs2289658 | rs2289657 | rs2289656 | rs3824519 |
| --- | --- | --- | --- | --- | --- | --- | --- |
| rs1439050 | 1 | 0.54 | 0.50 | -0.14 | -0.12 | 0.01 | -0.14 |
| rs1187352 |  | 1 | 0.75 | 0.04 | 0.11 | -0.06 | 0.14 |
| rs1778933 |  |  | 1 | 0.11 | 0.13 | -0.03 | 0.10 |
| rs2289658 |  |  |  | 1 | 1.00 | -1.00 | 0.79 |
| rs2289657 |  |  |  |  | 1 | -1.00 | 0.92 |
| rs2289656 |  |  |  |  |  | 1 | -1.00 |
| rs3824519 |  |  |  |  |  |  | 1 |
| rs56142442 |  |  |  |  |  |  |  |

*Linkage disequilibrium matrix present D’ criteria value between each SNPs. Moderate linkage disequilibrium was defined by D’ criteria absolute value between 0.50 and 0.75. High linkage disequilibrium was defined by D’ criteria absolute value between 0.75 and 1.*
