# Supplementary material for: The TRKB rs2289656 genetic polymorphism is associated with acute suicide attempts in depressed patients: A transversal case control study
Source: PLoS One. 2018 Oct 11;13(10):e0205648. doi: 10.1371/journal.pone.0205648 (PMC6181406; doi:10.1371/journal.pone.0205648)
Supplement: S5 Table — (DOCX) [file pone.0205648.s005.docx]

***S5 Table: Demographic and clinical features according to the TRKB alleles.***

|  | rs1439050 | | rs1187352 | | rs1778933 | | rs2289658 | | rs2289657 | | rs2289656 | | rs3824519 | |
| --- | --- | --- | --- | --- | --- | --- | --- | --- | --- | --- | --- | --- | --- | --- |
| Genotype | G | T | G | A | T | C | A | G | G | T | C | T | C | T |
| Allelic number | 653 | 411 | 755 | 341 | 775 | 327 | 1047 | 47 | 1065 | 41 | 861 | 233 | 1011 | 93 |
| Age (m(sd)) | 45,6 (13,2) | 47,4 (13,1) | 45,9 (13) | 46,9 (13,3) | 46,2 (13) | 46,5 (13,4) | 46,2 (13,2) | 47,7 (11,4) | 46,2 (13,1) | 48,5 (11,3) | 45,7 (13,3) | 48,2 (12) | 46,3 (13,2) | 45,7 (11,9) |
| Women (%(n)) | 70,8 (462) | 69,1 (284) | 70,5 (532) | 69,2 (236) | 69,7 (540) | 71,6 (234) | 71  (743) | 57,4 (27) | 70,3 (749) | 56,1 (23) | 69,6 (599) | 72,5 (169) | 70,1 (709) | 65,6 (61) |
| Single (%(n)) | 51,5 (336) | 54,5 (224) | 54  (408) | 51,6 (176) | 51,2 (397) | 56,6 (185) | 52,6 (551) | 53,2 (25) | 52,9 (563) | 51,2 (21) | 54,5 (469) | 46,8  (109) | 52,7 (533) | 50,5 (47) |
| High educational level (%(n)) | 46,2 (302) | 40,4 (166) | 44,2 (334) | 45,7 (156) | 44,1 (342) | 45,3 (148) | 44,6 (467) | 44,7 (21) | 44,6 (475) | 46,3 (19) | 46,2  (398) | 37,8  (88) | 44,2 (447) | 41,9 (39) |
| Smoking (%(n)) | 38,9 (254) | 34,1 (140) | 38,3 (289) | 33,7 (115) | 37  (287) | 35,2 (115) | 36,4 (381) | 36,2 (17) | 36,5 (389) | 41,5 (17) | 35,2 (303) | 41,6 (97) | 36,4 (368) | 43  (40) |
| Recurrent MDD (%(n)) | 75,3 (492) | 74,5 (306) | 74,1 (559) | 77,4 (264) | 73,5 (570) | 78,3 (256) | 75,5 (790) | 70,2 (33) | 75  (799) | 75,6 (31) | 75,6 (651) | 73,3 (171) | 74,8 (756) | 76,3 (71) |
| HAMD-17 (m(sd)) | 24,4 (4,9) | 24,7 (4,9) | 24,4 (4,9) | 24,8 (4,9) | 24,5 (4,9) | 24,6 (4,8) | 24,5 (4,9) | 24,8 (5,3) | 24,5 (4,9) | 24,8 (5,4) | 24,5  (5) | 24,5 (4,5) | 24,5 (4,8) | 24,5 (5,4) |
| Antidepressant drug free (%(n)) | 38,4 (251) | 35,8 (147) | 37,5 (283) | 36,1 (123) | 37,7 (292) | 36,1 (118) | 37,1 (388) | 38,3 (18) | 37,2 (396) | 34,1 (14) | 38,1 (328) | 33,5 (78) | 37  (374) | 36,6 (34) |
| Previous antidepressant treatment (%(n)) | 78,5 (513) | 78,6 (323) | 77,3 (584) | 81,5 (278) | 77,2 (598) | 81,7 (267) | 78,9 (826) | 76,6 (36) | 78,6 (837) | 80,5 (33) | 77,7 (669) | 81,9 (191) | 78,7 (796) | 79,6 (74) |
| Suicide Attempts |  | | | | | | | | | | | | | |
| Past (%(n)) | 31,2 (204) | 31,6 (130) | 31  (234) | 32,8 (112) | 30,2 (234) | 35,5 (116) | 31,9 (334) | 34  (16) | 31,8 (339) | 36,6 (15) | 32,5 (280) | 29,2 (68) | 31,2 (315) | 37,6 (35) |
| Acute (%(n)) | 22,1 (144) | 23,4 (96) | 22,6 (171) | 24,6 (84) | 23,2 (180) | 23,5 (77) | 23,2 (243) | 21,3 (10) | 23,3 (248) | 19,5  (8) | 25,7 (221) | 14,2 (33) | 22,8 (231) | 23,7 (22) |

*n: number of patient; m: mean; sd: standard deviation; HAMD-17: Hamilton Depression Rating Scale 17 items; antidepressant drug free: no antidepressant* since 3 years before assessment*; previous antidepressant treatment: past history of antidepressant treatment; MDD: Major Depressive Disorder; SA: Suicide Attempt; Past SA: Suicide attempts were defined as those which occurred more than one month before assessment.*
